# Association between Conflict and Cholera in Nigeria and the Democratic Republic of the Congo

**DOI:** 10.3201/eid2812.212398

**Published:** 2022-12

**Authors:** Gina E.C. Charnley, Kévin Jean, Ilan Kelman, Katy A.M. Gaythorpe, Kris A. Murray

**Affiliations:** Imperial College London, London, UK (G.E.C. Charnley, K.A.M. Gaythorpe, K.A. Murray);; Laboratoire MESuRS–Cnam Paris, Paris, France (K. Jean);; Institut Pasteur, Paris, France (K. Jean); University College London, London (I. Kelman);; University of Agder, Kristiansand, Norway (I. Kelman);; MRC Unit The Gambia at London School of Hygiene and Tropical Medicine, Fajara, The Gambia (K.A. Murray)

**Keywords:** cholera, public health, epidemiology, disease outbreaks, vibrio cholerae, conflicts, Nigeria, Democratic Republic of the Congo, bacteria

## Abstract

Cholera outbreaks contribute substantially to illness and death in low- and middle-income countries. Cholera outbreaks are associated with several social and environmental risk factors, and extreme conditions can act as catalysts. A social extreme known to be associated with infectious disease outbreaks is conflict, causing disruption to services, loss of income, and displacement. To determine the extent of this association, we used the self-controlled case-series method and found that conflict increased the risk for cholera in Nigeria by 3.6 times and in the Democratic Republic of the Congo by 2.6 times. We also found that 19.7% of cholera outbreaks in Nigeria and 12.3% of outbreaks in the Democratic Republic of the Congo were attributable to conflict. Our results highlight the value of providing rapid and sufficient assistance during conflict-associated cholera outbreaks and working toward conflict resolution and addressing preexisting vulnerabilities, such as poverty and access to healthcare.

Diarrheal diseases are the eighth leading cause of death worldwide; cholera contributes substantially, especially in low- and middle-income countries (*1*). Among cases reported by the World Health Organization (WHO), >94% are in Africa (*2*). Previous research has found several environmental and socioeconomic links with cholera, including temperature; precipitation; poverty; and water, sanitation, and hygiene (WASH) (*3*,*4*). Furthermore, extremes of these environmental and social conditions (e.g., droughts, floods, conflicts) can act as catalysts for outbreaks (*4*–*6*).

We focused on the effects of conflict on cholera outbreaks and compared the results for 2 countries in Africa, Nigeria and the Democratic Republic of the Congo (DRC), over the past 23 years. Several mechanisms through which conflict can lead to infectious disease outbreaks have been suggested (*7*–*9*). During conflicts, services can be disrupted, including access to WASH, disruption of disease control programs, and collapse of health systems (e.g., vaccination coverage). Persons displaced by conflict may also find it difficult to access healthcare (*10*–*12*). Populations may not seek medical treatment because they perceive healthcare facilities as unsafe. For example, during the 2018 Ebola outbreak in DRC, healthcare facilities were attacked, dampening efforts to control the virus (*12*). Conflict can worsen preexisting vulnerabilities, including poverty, because conflicts can cause loss of income, disruption to education, damage to livelihoods, and displacement (*13*).

Nigeria and DRC have social and environmental similarities as well as cholera outbreaks. Both countries experience active conflicts, such as the Boko Haram insurgency in northeastern Nigeria (*14*) and political unrest in eastern DRC (*15*). They also have the second (Nigeria) and third (DRC) highest numbers of estimated cholera cases per year in Africa (*16*); the most active cholera foci in the world are the DRC Kivu provinces (*17*). In addition, known cholera risk factors are present in Nigeria and DRC: tropical climate; poor access to WASH; and a large proportion of the population living in poverty (<$1.25/day), 87.7% for the DRC and 62% for Nigeria (*18*).

Few studies have investigated the effects of conflict on cholera outbreaks, especially quantitatively. Studies have commonly focused on cholera and conflict in Yemen (*8*,*19*), the effects of conflict on vaccination efforts (*20*), or the effects of conflict on other diseases such as Ebola (*12*) and COVID-19 (*21*). Despite reporting a large proportion of global cases, Africa is a chronically understudied continent with regard to cholera (*2*).

To bridge this research gap, we used the self-controlled case series (SCCS) method, nationally and subnationally, and to provide insight into the effects of lag and cholera definition, we completed a sensitivity analysis. We used the SCCS method in a novel application and aim to explore and promote its use in other contexts (*22*). Previous uses include testing the effectiveness of drug and vaccine interventions at the individual (*23*,*24*) and population levels (*25*). Furthermore, to determine the proportion of cholera outbreaks attributable to conflict, we adapted the recently developed percentage attributable fraction (PAF) equations to this study (*25*). On the basis of these results, we suggest mechanisms for which conflict is driving cholera and potential risk factors, building on previous research in this area. We hope this information can be used to strengthen disease prevention in conflict settings and reduce additional illness and death during conflicts.

## Methods

### Datasets

We compiled cholera data from a range of publicly available sources: WHO disease outbreak news, ProMED, ReliefWeb, WHO Regional Office for Africa weekly outbreak and emergencies, UNICEF cholera platform (https://www.unicef.org), EM-DAT (https://emdat.be), the Nigerian Centre for Disease Control, and a literature search in English and French. The data are available in a GitHub repository (https://github.com/GinaCharnley/cholera_data_drc_nga), and additional information on data collation and validation are available in a complementary database paper (*26*). An outbreak was defined by the onset of the first cholera case, and the case definitions for the 2 countries are shown in the Appendix. Conflict data were provided by the United Nations Office for the Coordination of Humanitarian Affairs Humanitarian Data Exchange, which provides data from the Armed Conflict Location and Event Data Project (*27*). The data included subnational conflicts, categorized by type (e.g., battles, explosions, protests, riots, strategic developments, and violence against civilians).

The spatial granularity of the analysis was to administrative level 1 (states for Nigeria and provinces for DRC), and we aggregated all data points that were reported on a finer spatial scale to the upper level. The study period was January 1997–May 2020, the dates of the first and last reports in the conflict datasets. The temporal scale was set to weekly, with continuous weeks from epidemiological week 1 in 1997 through epidemiologic week 20 in 2020 (1–1,220 continuous weeks). We chose continuous weeks to be compatible with the model and to include periods of conflict that endured from one year into the next. We chose weeks, rather than days, to account for reporting lags because previous work has reported issues in the granularity of data and timeliness of reporting, especially during humanitarian crises, because of different sources of data and logistical difficulties (*28*,*29*) (Appendix).

### Model Structure and Fitting

The SCCS method investigates the association between an exposure and an outcome event. The aim of SCCS is to estimate the effect, by comparing the relative incidence of the adverse events (outbreaks) within an exposure period of hypothesized excess risk (conflicts), compared with all other times (peace, according to the dataset used). The SCCS method is a case-only method and has the advantage of not needing separate controls by automatically controlling for fixed confounders that remain constant over the observation period (*30*,*31*).

Both the exposure and the event were set as binary outcomes, either being present (1) or not (0). The observation period was the full study period (1–1,220 continuous weeks). The exposure period was the first week after conflict onset and was reported as multiple onsets for each event, not 1 long exposure period incorporating all events in the specific week (or 2, 4, 6, 8, and 10 weeks). The event was defined by the week the cholera outbreaks were reported. Each event and exposure that occurred in the same state/province were assigned an identification number and a preexposure, exposure, and postexposure period (Appendix Table).

We fit the data to conditional logistic regression models by using the event (cholera outbreak onset) as the outcome variable [function clogit() in the R package survival] (*32*). As is standard for conditional logistic regression, the interval between the exposure to nonexposure period was offset (coefficient value of 1) in the model and the identification numbers were stratified. The model coefficient values were used to calculate incidence rate ratio (IRR), which quantifies the magnitude to which conflict increased the rate of cholera outbreaks.

To determine whether the significance of the effect of conflict on cholera outbreaks varied by subnational location and whether conflict was more influential in some states/provinces than others, we next split the datasets for each country by state/province and repeated the analysis for each. We conducted all statistical analyses by using R version 3.6.2 (The R Project for Statistical Computing, https://www.r-project.org), and the threshold for significance was p<0.05.

### Sensitivity Analysis

We used a sensitivity analysis to test different methods of defining the exposure end point, which was set to 1 week in the main analysis and 2, 4, 6, 8, and 10 weeks in the sensitivity analysis. Our aim was to further determine how long after conflict exposure the rate of cholera was heightened (Appendix Figures 1, 2). 

To determine the effect of altering the cholera outbreak definition and to test for the temporal autocorrelation, we completed an additional sensitivity analysis that involved 2 scenarios. Scenario 1 removed all outbreaks within 2 weeks of each other (based on cholera biology: up to 10 days for bacterial shedding plus up to 5 days for incubation period) (*33*,*34*). Scenario 2 was an extreme scenario to fully test model robustness and removed all outbreaks within 6 months of each other.

### PAF

We adapted the recently developed PAF equations (*30*) to the model output and data (Appendix). The PAF values estimate the percentage of outbreaks that could be attributed to conflict at a national level, and we used the full observation period of the datasets and the IRR values from the model results. We used bootstrap resampling (1,000 samples) to obtain 95% CIs For each sample, we randomly sampled a value of IRR according to the parameters estimated in the SCCS analysis.

## Results

### Conflict and Cholera Occurrence

**Figure 1:**
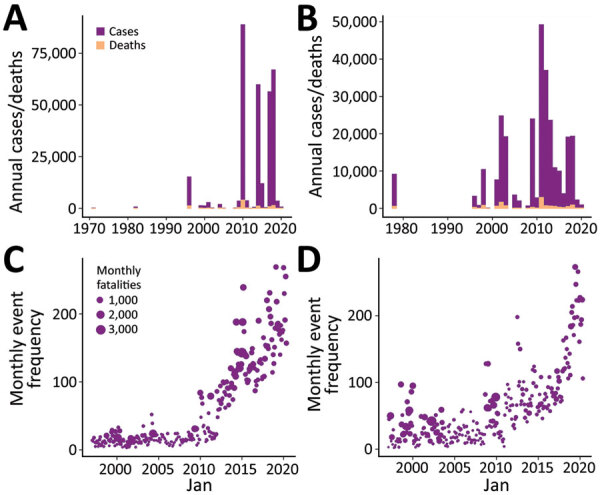
Changes in cholera and conflict for the full datasets used in study of the association between conflict and cholera in Nigeria and the Democratic Republic of the Congo (DRC). A, B) Monthly cholera cases and deaths for Nigeria (A) and DRC (B). C, D) Monthly frequency of conflict exposures and fatalities for Nigeria (C) and DRC (D).

**Figure 2:**
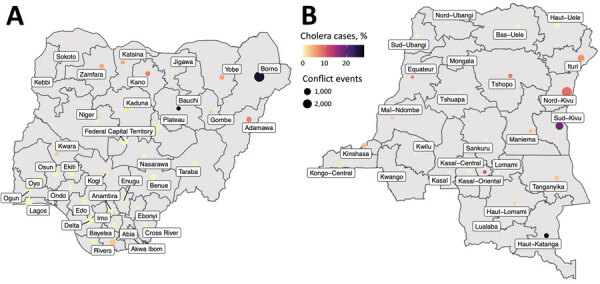
Number of conflicts and cholera cases as a percentage of the total number of national cases by administrative level 1 for Nigeria (A) and the Democratic Republic of the Congo (B).

Temporal and spatial data showing the distribution of conflict and cholera in Nigeria and the DRC show an increase in reported conflict and cholera, especially after 2010 (Figure 1, panels A–D). A large proportion of the cholera cases have been reported in conflict-stricken areas (Figure 2).

**Figure 3:**
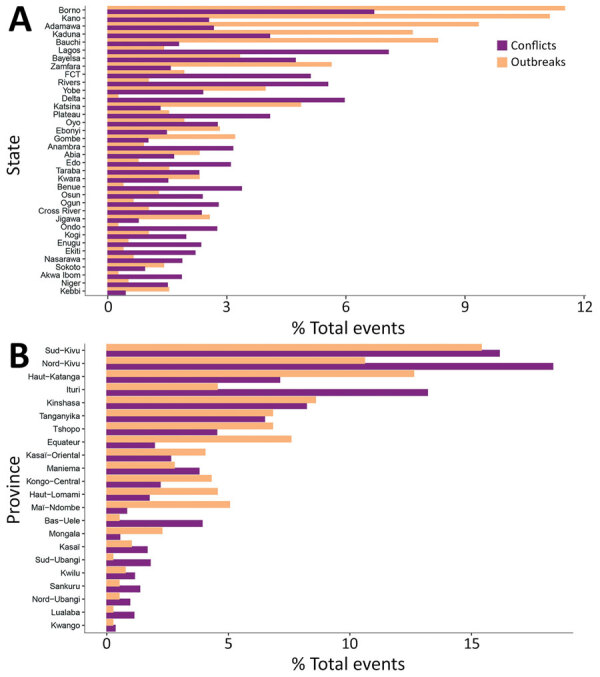
Percentage of events in each dataset used in study of the association between conflict and cholera for Nigeria (A) and the Democratic Republic of the Congo (B) by administrative level 1. FCT, Federal Capital Territory.

The total number of conflicts and outbreaks for each state/province during the study period totaled 8,190 conflicts and 782 cholera outbreaks for Nigeria and 4,639 conflict and 396 cholera outbreaks for DRC (Figure 3). The outbreak distribution applied satisfactorily to the Poisson probability distribution (Appendix Figure 3).

**Figure 4:**
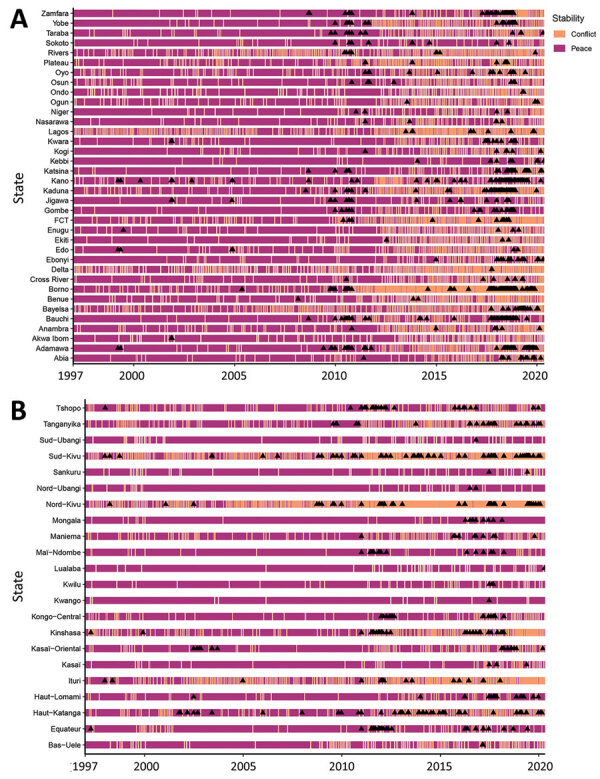
Swimmer plots showing the conflict exposure period in the self-controlled case series model (1 week after the onset) and the outbreaks (black triangles) for each state/province for Nigeria (A) and the Democratic Republic of the Congo (B). Data were compiled by epidemiologic week. FCT, Federal Capital Territory.

To be included in the analysis, a state/province had to report outbreaks and conflicts during the study period; because the SCCS method is a case-only approach, we excluded states/provinces that reported only conflicts (not any outbreaks). As such, 36 states were included for Nigeria and 22 provinces for DRC (Figure 4; Appendix). 

### Model Output

Conflict significantly increased the rate of cholera outbreaks (IRR) in the past 23 years in Nigeria and DRC (p<0.05). The effect was of greater magnitude in Nigeria, increasing the risk for cholera outbreaks by up to 3.6 times (IRR 3.6 times, 95% CI 3.3–3.9 times), whereas, for DRC, the risk was increased by 2.6 times (IRR 2.6 times, 95% CI 2.3–2.9 times).

**Figure 5:**
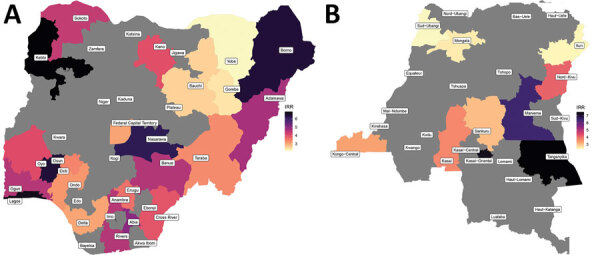
IRRs for the effect of exposure to conflict within 1 week of the event and cholera at a subnational level for Nigeria (A) and the Democratic Republic of the Congo (B). Only results that were significant at the threshold p<0.05 are plotted. IRR, incidence rate ratio.

Of the 36 Nigeria states included in the analysis, we found statistically significant associations between conflict and cholera outbreaks for 24. The strongest effects were in Kebbi, Lagos, Osun, Borno, and Nasarawa; IRR values ranged from 6.2 to 6.8 times (Figure 5, panel A).

Of the 22 DRC provinces included in the analysis, we found a statistically significant relationship between conflict and cholera for 11. The strongest values were for Tanganyika, Kasaï-Oriental, Maniema, Nord-Kivu and Kasaï, and some were the highest values in the analysis. In Tanganyika, conflict increased cholera outbreak rate by 7.5 times and in Kasaï by 3.7 times (Figure 5, panel B).

### Sensitivity Analyses

The effect of conflict on cholera outbreaks at the national and subnational level for Nigeria and DRC decreased with increasing exposure period. The decrease in IRR from week 1 to week 10 was from 3.6 to 2.08 for Nigeria and 2.6 to 1.5 for DRC. By week 6, the change was minimal and plateaued or increased (Appendix Figures 4, 5).

Changing the outbreak onset definition yielded results similar to those of the original analysis. Removing events within 2 weeks and within 6 months of each other led to IRR values within the 95% CI of the initial definition. All results remained significant at p<0.05 and provide evidence that temporal autocorrelation did not affect model robustness (Appendix Figure 6).

### PAF

The IRR values from the model results indicating 3.6 for Nigeria and 2.6 for DRC were randomly resampled (1,000 samples). On the basis of these results, the onset of a conflict during the period from epidemiologic week 1 in 1997 to week 20 in 2020 was attributable to 19.7% (95% CI 18.2%–21.2%) of cholera outbreaks in Nigeria and 12.3% (95% CI 10.2%–14.4%) in DRC.

## Discussion

Conflict was associated with an increased rate of cholera outbreaks by 3.6 times in Nigeria and 2.6 times in DRC. The percentages of cholera outbreaks attributable to conflicts during 1997–2020 (1,220 continuous weeks) were 19.7% for Nigeria and 12.3% for the DRC. The states/provinces where risk was highest were Kebbi, Nigeria, at 6.9 times, and Tanganyika, DRC, at 7.3 times. This finding shows that the effect of conflict was much greater in some states/provinces than at the national level.

The sensitivity analysis evaluating the effect of lag showed decreasing effect as the weeks progressed; in some states/provinces, the effect plateaued or increased around 6 weeks after the exposure. The decrease with the lag duration may be a diluting effect because the probability of an outbreak will increase across a longer period. The states/provinces that increased after week 6 were often those with the strongest initial effect, especially in the DRC. The larger initial effect having a longer lasting effect may potentially result from conflict severity. The IRR values remained at >1 (2.08 Nigeria and 1.5 for DRC) at 10 weeks after the conflict, providing further evidence of a long-lasting effect of conflict.

States/provinces where rates of cholera increased most often coincided with areas of high conflict. This association further supports the hypothesis that conflict may be a driver of cholera in Nigeria and DRC. The effect of conflict exposure on cholera was also highly significant in states/provinces surrounding high-conflict areas (e.g., Abia, Ogun, Osun, Maniema, and Tanganyika), showing a potential spillover effect. The states/provinces were studied independently, but a possible explanation may be the fleeing of persons from areas of conflict or a cholera outbreak to neighboring states, because displacement is a known risk factor for disease outbreaks (*9*). This explanation is relevant for cholera because a large proportion of persons can be asymptomatic but still shed the pathogen into local reservoirs, which other persons use as drinking water because of a lack of alternatives (*33*).

Cholera outbreaks can be explosive and self-limiting because of the high number of asymptomatic persons, diluting the pool of susceptible persons (*33*), potentially explaining why the effects of conflict on cholera were seen just 1 week after the event. The incubation period of cholera is short (*34*), making the effect within the first week found here biologically possible for the pathogen and the time frame for elevated exposure realistic for resulting in cases. Other examples of cholera cases emerging within the first week after an adverse event include Cyclone Thane in the Bay of Bengal (*35*), water supply interruption in DRC (*36*), and Cyclone Aila in West Bengal, India (*37*). These examples provide further evidence of the need for quick and effective aid during humanitarian crises to avoid outbreaks and reduce deaths (*38*).

During periods of conflict, healthcare facilities can suffer and cholera outbreaks can overwhelm systems, potentially leading to the association between conflict and cholera. Care can be inaccessible because of direct infrastructure damage or difficulties getting to the facilities because of impromptu roadblocks (*39*). Supplies may be stolen or not deliverable, including oral rehydration solution, pathogen-sensitive antimicrobial drugs, and oral cholera vaccines, all of which are needed during cholera outbreaks (*40*). Last, safety is a serious concern for healthcare workers and patients; nongovernmental organizations can withdraw from these areas, citing an inability to ensure the safety of their staff (*41*). Steps need to be taken globally to reduce violence against healthcare workers, such as using active clinical management for all patients to enhance the acceptance of pathogen-specific treatment centers (*42*).

Conflict has the potential to worsen preexisting vulnerabilities, which can exacerbate poverty, another potential cause of the effect of conflict on cholera. The effects of poverty can be far-reaching and are a known risk for cholera (*4*,*43*) along with other diseases (*44*). For example, because of crowding and poor access to WASH, poor urban settlements have faced the brunt of outbreaks, including Zika infection, Ebola virus disease, typhoid, and cholera (*45*). Conflict can result in loss of possessions, loss of habitual residence, and an inability to find employment, thereby reducing income generation, savings, and financial backstops (*13*). In times of worsening poverty, persons may not be able to afford healthcare and basic medical supplies, especially those in vulnerable groups. This disruption to daily life can cause many more deaths than direct battlefield fatalities and leads to stagnated development (*46*).

Although we did not directly evaluate WASH and poverty, a lack of WASH facilities is likely to have contributed to the positive association between cholera and conflict. Conflict can lead to disruption in sanitation and hygiene, and adverse events can act as catalysts in the interaction of contaminated water and the human populations (*3*). Displacement from conflict can cause difficulties accessing WASH (e.g., latrine access, soap availability), and rapid cholera outbreaks have occurred in several displacement camps, including in DRC after the Rwanda genocide in 1994 (*2*). Displacement of persons because of conflict may result in the use of water contaminated with toxigenic strains of *Vibrio cholerae* because alternative water sources are lacking, leading to outbreaks.

A potential limitation of our analysis is the plausible existence of multiple causal pathways, leading to misclassification because of time/variant confounders. Examples include a conflict in an adjacent geographic area being causally linked to the conflict in the current geographic area or the presence of bodies of water, which are considered fundamental in cholera transmission (*47*,*48*). Additional environmental factors (e.g., seasonal weather changes and preexisting vulnerabilities) are beyond the scope of the methods that we used, which investigate conflict in isolation.

The degree of effect that we found may be affected by underreporting, overreporting, and delayed reporting. Underreporting is a significant issue in global cholera and conflict estimates because of asymptomatic case-patients, disincentives to report, and logistics issues (*29*,*49*). Cholera surveillance is difficult during conflicts because of displaced populations and security concerns. In addition, our method may have resulted in a classification bias, underestimating the effect of conflict on cholera. If a cholera outbreak was imported from a neighboring state/province (spatial autocorrelation), it would be classified as a genuine, autochthonous event, which would probably be nondifferential (likely to happen during a period of exposure or nonexposure). Alternatively, during times of conflict, health surveillance can be enhanced by the government or nongovernmental organizations. Reporting delay is another potential problem, and some national reporting delays have been found to range from 12 days for meningococcal disease to 40 days for pertussis (*28*).

The SCCS model is a case-only approach; analyzing cases only, instead of the corresponding complete cohort, results in loss of efficiency. However, previous work has shown that the loss is small, especially when the fraction of the sample experiencing the exposure is high (Appendix). Moreover, loss of efficiency must be weighed against better control of time-invariant confounders. Previous examples illustrated that the SCCS design is likely to produce more trustworthy results than the corresponding cohort analysis, especially when a strong residual confounding bias is likely (*30*,*31*).

We did not evaluate the severity or intensity of the conflict and cholera outbreaks; instead, we used a binary variable. Conflict severity is complex, far-reaching, and challenging to measure. Making assessments and assumptions of how conflict affects a health outcome is difficult and may involve oversimplification. Qualitative conflict severity research is needed but is beyond the scope of this article. 

Despite the limitations of conflict and cholera data, the data that we used are of the highest standard available and have been used by several other studies, making the research comparable (*11*,*12*). In addition, we used several methods to validate the cholera data (*26*). Creating partnerships with those working on the ground and exploring more sensitive data options is an area of future research. Additional methods that we used to account for data limitations included setting both the event and the exposure to a binary outcome to reduce the effects of severity and using a weekly instead of daily temporal scale to account for delays.

In summary, our analysis shows a clear relationship between cholera and conflict in Nigeria and DRC; conflict was associated with an increased rate of cholera by up to 7.3 times in some states/provinces. The flexibility of SCCS and conditional logistic regression models makes future work evaluating different diseases, countries, and additional risk factors relatively simple. Cholera risks are probably multifactorial and complex; however, sufficient and rapid support, along with enhanced efforts to build community trust can reduce this excess risk. Finding conflict resolution and addressing preexisting vulnerabilities (poverty, healthcare, and WASH) should be the main priority. Reducing those vulnerabilities will give communities greater resources to adapt and reduce vulnerabilities in times of conflict as well as peace.

Appendix Additional information for study of the association between conflict and cholera in Nigeria and the Democratic Republic of Congo.
