# Supplementary material for: Association between Conflict and Cholera in Nigeria and the Democratic Republic of the Congo
Source: Emerg Infect Dis. 2022 Dec;28(12):2472–81. doi: 10.3201/eid2812.212398 (PMC9707578; doi:10.3201/eid2812.212398)
Supplement: Appendix — Additional information for study of the association between conflict and cholera in Nigeria and the Democratic Republic of Congo. [file 21-2398-Techapp-s1.pdf]

Article DOI: https://doi.org/10.3201/eid2812.212398

Association between Conflict and Cholera in Nigeria and the Democratic Republic of the Congo

Appendix

Cholera Case Definitions According to the Nigerian Centre for Disease Control and the Ministère de la Santé Publique de la République Démocratique du Congo

NCDC:

Suspected case: Severe dehydration or death from acute watery diarrhoea in a patient aged 5 years or more. In an epidemic situation: A suspected case in any person aged 5 years or more with acute watery diarrhoea with or without vomiting.

Confirmed case: A suspected case in which *Vibrio cholerae* O1 or O139 has been isolated in the stool.

RDC Ministère de la Santé:

Suspected case: Severe dehydration or death following acute watery diarrhoea in a patient aged 5 years or more. In an epidemic situation: Acute watery diarrhoea with or without vomiting in a patient aged 1 year or more.

Dataset Information

Cholera data was compiled from a range of publicly available sources (WHO's disease outbreak news, ProMED, ReliefWeb, WHO's regional office for Africa weekly outbreak and emergencies, UNICEF cholera platform, EM-DAT, the Nigerian CDC and a literature search) in both English and French. A data charting form was used to enable a dynamic data entry process and collected data on date, geographic location, cases, deaths, hospitalisations, fatality rates, gender, age, oral cholera vaccinations, risk factors, aid and the source of the report. Data spanned

from 1971-2021 for Nigeria and 1978-2021 for the DRC on a daily temporal scale and was provided at the finest spatial scale possible.

Conflict data was provided by the United Nations Office for the Coordination of Humanitarian Affairs's Humanitarian Data Exchange (HDX, 2020). The data included sub-national conflict events for both countries on a fine spatial scale, given to the exact location in longitude/latitude. This was reported on a daily temporal scale and spanned from 1997 to 2020. The data was also categorised by event type which included battles, explosions, protests, riots, strategic developments and violence against civilians. This was further sub-categorised within these groups and reported number of fatalities.

The study period was selected as Jan 1997 to May 2020, as these were the first and last reports in the conflict data. The spatial granularity of the analysis was to administrative level 1 (states for Nigeria and provinces for the DRC) and all data points that were reported on a finer spatial scale were attributed to the upper level. To be included in the analysis, the state/province had to report both outbreaks and conflicts during the study period, therefore 22 provinces were included for the DRC and 36 states for Nigeria.

Sensitivity Analysis

Alternative exposure end points to identify the effect of lag.

Five alternative exposure periods were tested from the original exposure period (1 week after the onset of exposure, lag 1) and were named lag periods due to the potential lag effect from conflict onset to cholera outbreaks, these included:

1. Lag 2 - Week of conflict onset + 2 weeks
2. Lag 4 - Week of conflict onset + 4 weeks
3. Lag 6 - Week of conflict onset + 6 weeks
4. Lag 8 - Week of conflict onset + 8 weeks
5. Lag 10 - Week of conflict onset + 10 weeks

The sensitivity analysis was run on both a national and sub-national level and S1 and S2 Figs show additional swimmer plots of lag 10 and line plots of the temporal trends.

Equations Used to Calculate the Percentage Attributable Fraction

First the number of outbreaks attributable to conflicts, A_i, for each province i. Is estimated using the formula:

$$A_i = \lambda_i d_i^{E+}(IRR - 1) \quad (1)$$

Where d_i^{E+} is the total duration of conflict exposure for the province i (if no conflict in province i, thus $d_i^{E+} = 0$), λ_i is the rate of outbreak occurrence in a Poisson process in the absence of conflict, and IRR is the incidence rate ratio associated with exposure to conflict. With N_i^{E-} being the number of outbreaks observed in the province i during the un-exposed period and T being the total period of observation, an estimator of λ_i is $\hat{\lambda}_i = N_i^{E-}/(T - d_i^{E+})$, which leads to:

$$\hat{A} = \sum_i \frac{N_i^{E-} d_i^{E+}}{(T - d_i^{E+})}(\hat{IRR} - 1) \quad (2)$$

Based on $\hat{A}$ and N, the total number of outbreaks observed, we can easily obtain the equivalent of the population attributable fraction, PAF, which corresponds to the proportion of the total number of outbreaks in both countries that are attributable to conflicts (this is equivalent to the PAF obtained in classical epidemiological studies, but here population refers to the "population of provinces"):

$$PAF = \frac{\hat{A}}{N} \quad (3)$$

Excluded Events

States/provinces removed as they did not report conflict and cholera in the study period (1997-2020).

Democratic Republic of Congo:

. Haut-Uele - 629 conflict events removed

. Kasaï-Central - 234 conflict events removed

. Lomani - 101 conflict events removed

. Tshuapa - 70 conflict events removed

Nigeria:

. Imo - 239 conflict events removed

The data (datLong) was fit to the model as follows: clogit(event ~ exgr + strata(indiv) + offset(loginterval), data = datLong). The data set up follows the work of Heather Whittaker, further code and examples are available at: http://stats-www.open.ac.uk/sccs/r.htm. The data are based on the examples related to multiple risk periods. The aim is to evaluate the likelihood of event = 1 and exgr = 1, vs event = 1 and exgr = 0. A pre and post exposure period are included to account for the possibility that the event could increase or decrease the probability of an exposure and because exposures can occur after the event. The interval is set up as an offset to account for that fact that a longer interval would increase the chances of the event occurring within it, not because the exposure increased the event but because there was a greater period of time for it to occur by chance.

Additional explanations of these assumptions are available at: 1, Petersen I, et al. Self controlled case series methods: an alternative to standard epidemiological study designs. BMJ 2016;354. 2, Farrington CP, et al. Case series analysis for censored, perturbed, or curtailed post-event exposures. Biostatistics 2009;10(1):3-16.

Appendix Table. The layout of the pseudo-dataset dataframe fitted to the model. Each event and exposure are given a reference number (indiv).

indiv	exday	eventday	start	end	event	exgr	interval	loginterval
1	3	374	1	3	0	0	2	0.693147
1	3	374	3	4	0	1	1	0
1	3	374	4	542	1	0	538	6.287859
2	4	374	1	4	0	0	3	1.098612
2	4	374	4	5	0	1	1	0
2	4	374	5	542	1	0	537	6.285998

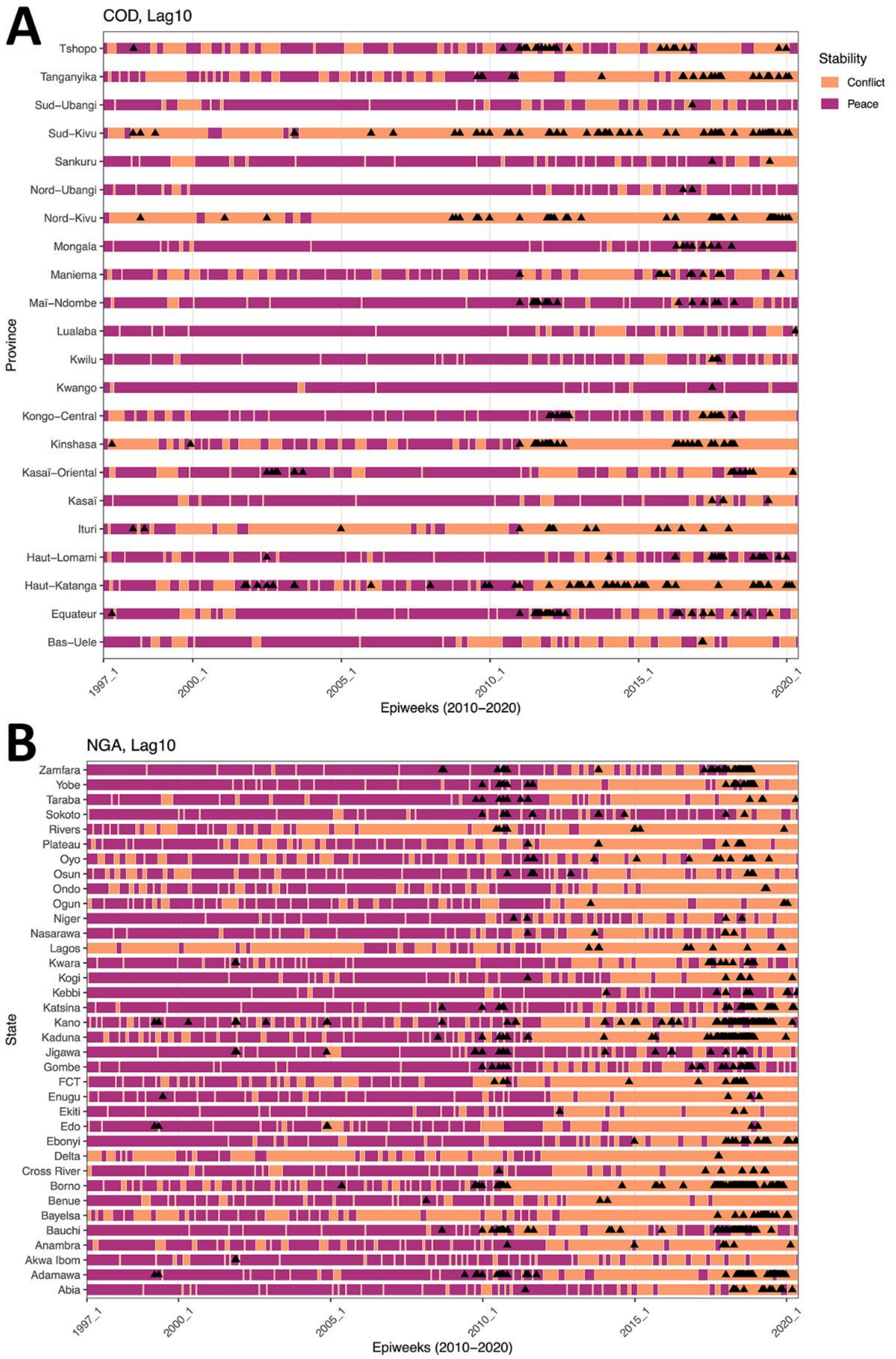

Appendix Figure 1. Swimmer plots showing the conflict dataset for lag 10 in the sensitivity analysis. In relation to outbreaks (black triangles) for Nigeria (NGA) and the Democratic Republic of Congo (COD).

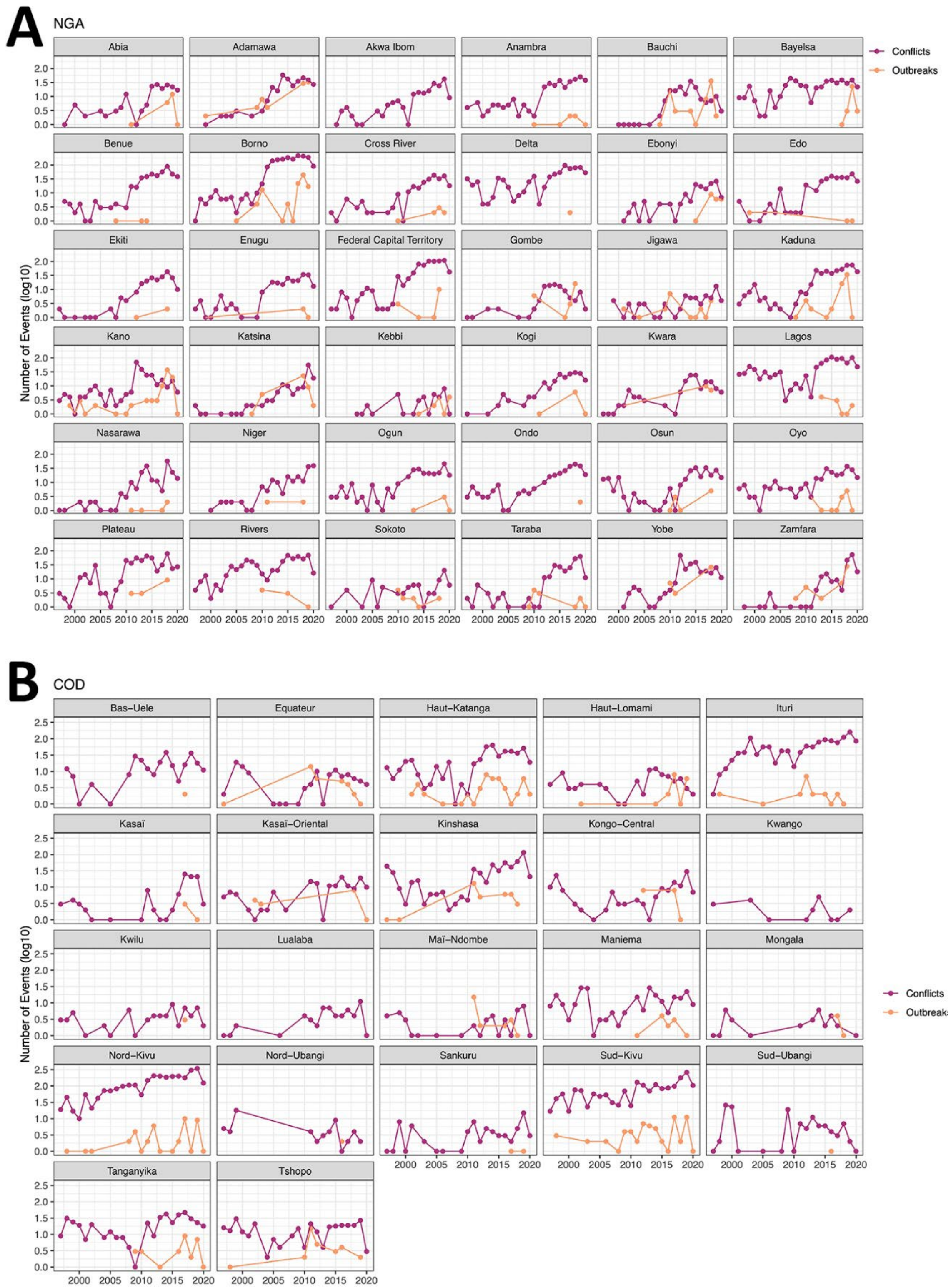

Appendix Figure 2. Number of outbreak (orange) and conflict (purple) events by year in Nigeria and the Democratic Republic of Congo over the full study period.

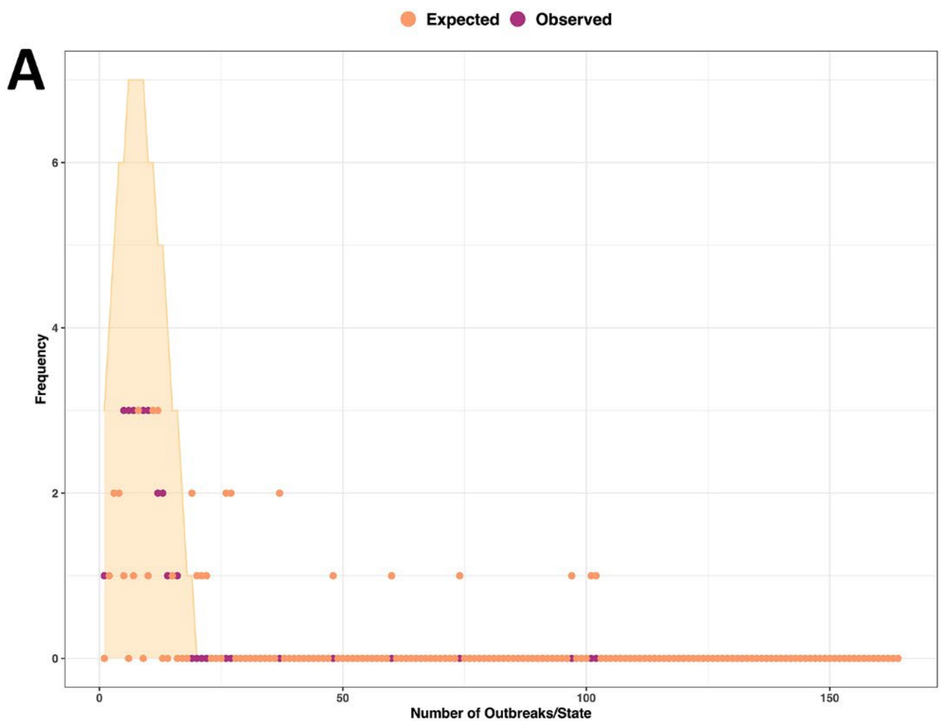

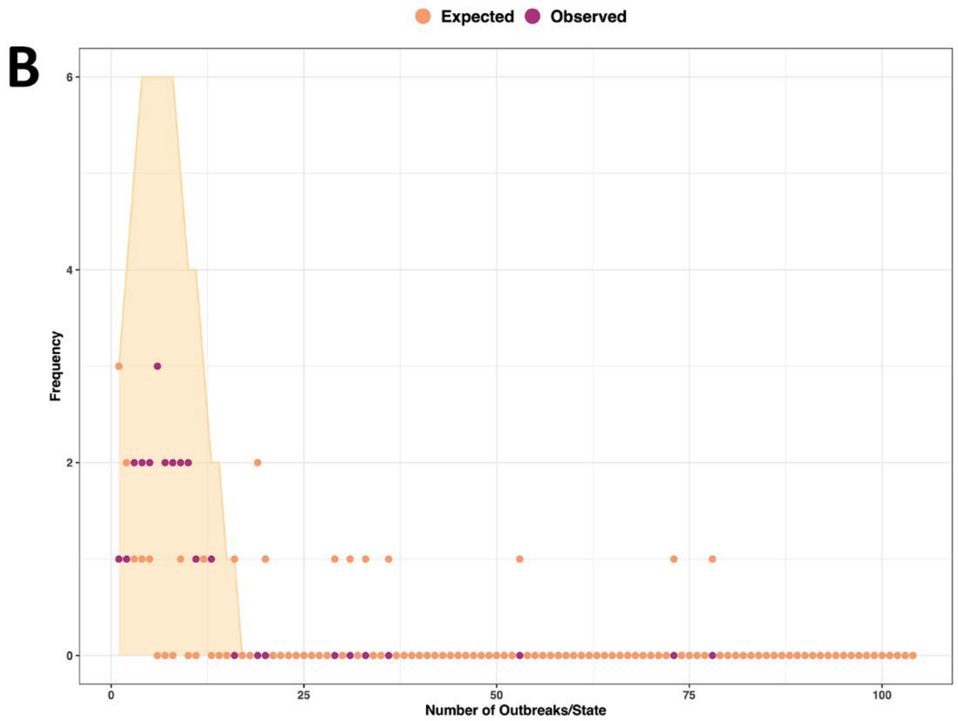

Appendix Figure 3. Poisson probability distribution fit to the outbreak data. The simulated counts were obtained from 10,000 random realizations of a Poisson process of rate λ = number of total national outbreaks/number of states or provinces, for **a,** Nigeria and **b**, the Democratic Republic of Congo. Expected values are the median simulated counts from the distribution with a 95% confidence interval.

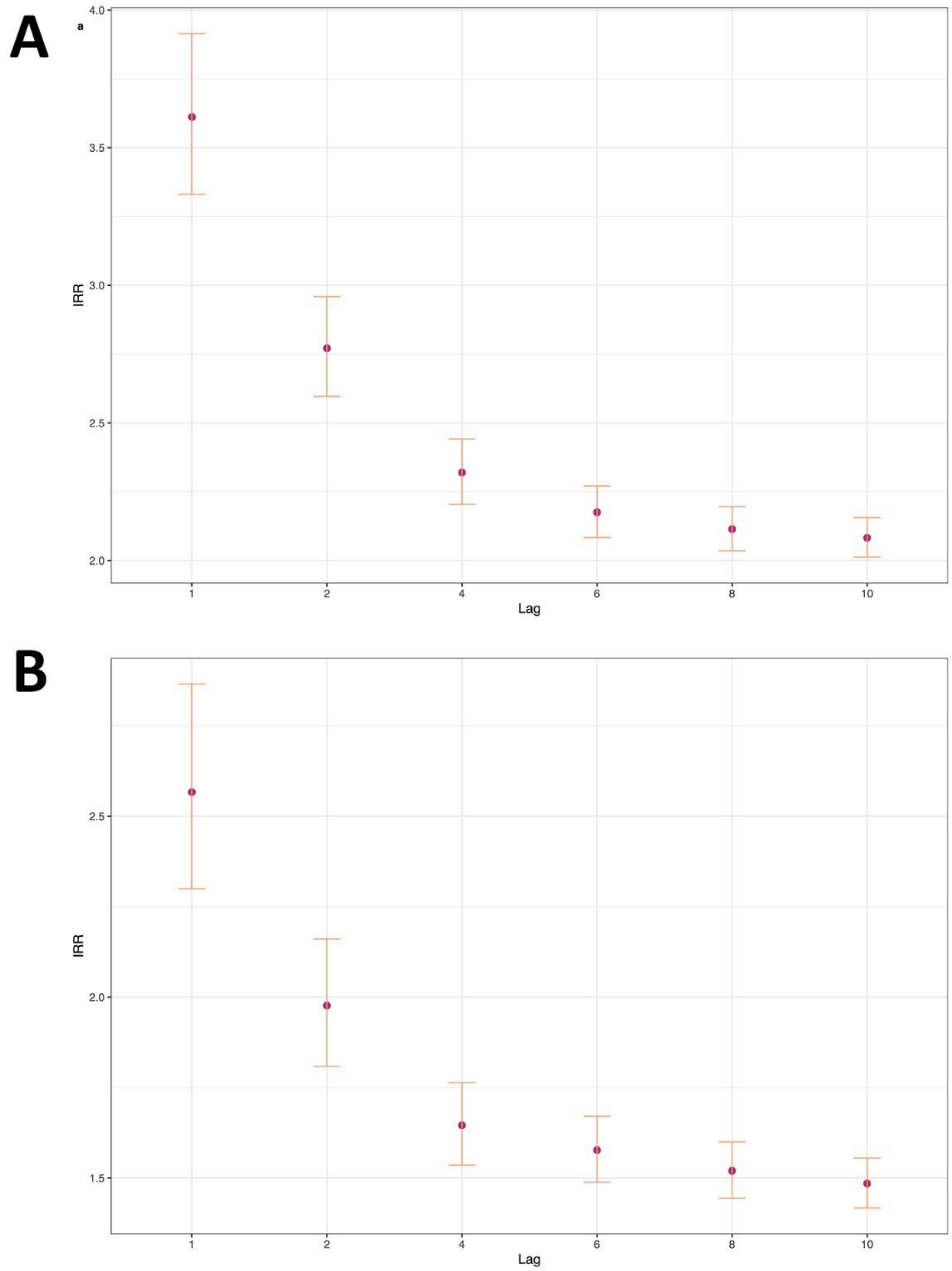

Appendix Figure 4. Results of national lag period sensitivity analysis. Incidence rate ratio (IRR) for the effect of exposure to conflict within 1, 2, 4, 6, 8 and 10 weeks of the event and cholera for **a**, Nigeria and **b**, the Democratic Republic of Congo. Only results that were significant at the threshold $p \leq 0.05$ are plotted here. From week 1 to week 10 the risk decreased from 3.6 to 2.08 for Nigeria and from 2.6 to 1.5 for the DRC. This suggests that the risk of conflict on cholera is highest soon after the event but remains a detectable association albeit at a lower level for potentially a long period of time after the event.

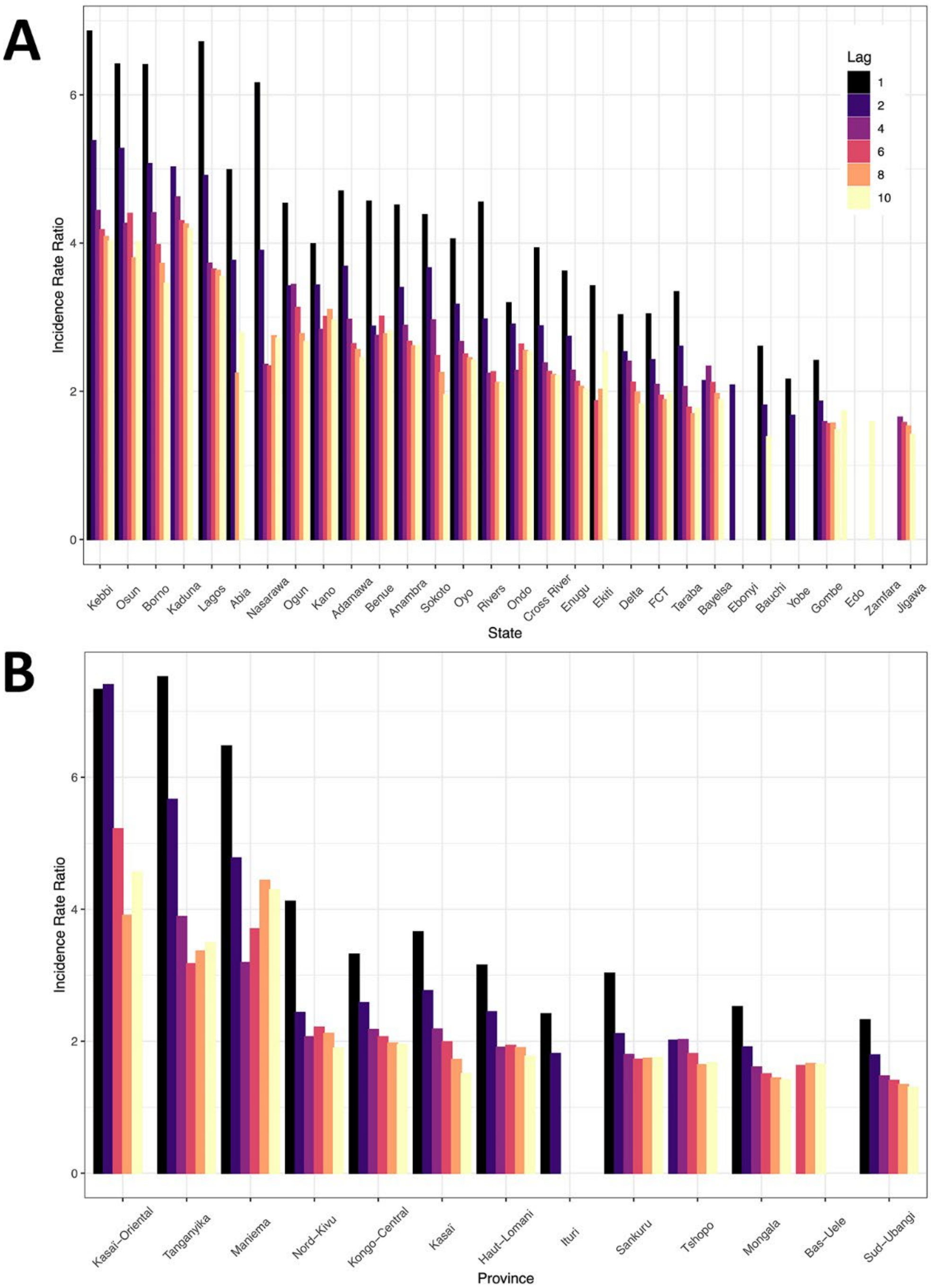

Appendix Figure 5. Results of subnational lag period sensitivity analysis. Incidence rate ratio (IRR) for the effect of exposure to conflict within 1, 2, 4, 6, 8 and 10 weeks of the event and cholera at administrative level 1. For **a**, Nigeria and **b**, the Democratic Republic of Congo. Only results that were significant at the threshold $p \leq 0.05$ are plotted here. Thirty Nigerian states and 13 DRC provinces were found to be significant for at least one of the lag periods and the most significant states predominately followed the trends of the national analysis. Values ranged from Kebbi at 6.9 to 4.0 times increased risk of cholera, to Gombe at 2.4 to 1.5.

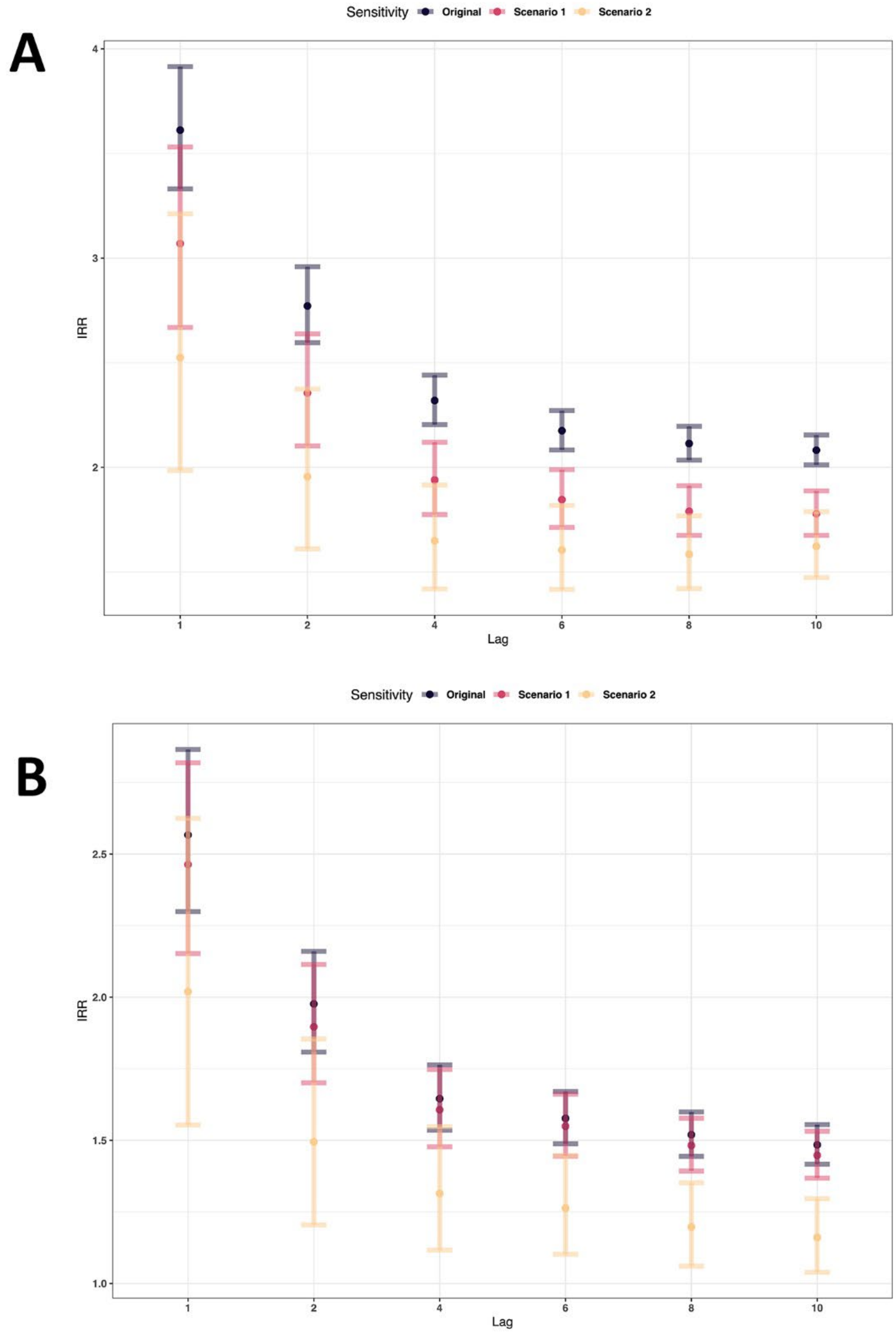

Appendix Figure 6. Results of outbreak definition sensitivity analysis. Incidence Rate Ratio (IRR) values and 95% confidence interval for **a**, Nigeria and **b**, the Democratic Republic of Congo for Scenario 1 removing all outbreaks within 2 weeks of each other (10 days shedding + 5 days incubation) and Scenario 2 removing all outbreaks within 6 months of each other. Both alternative scenarios are compared against the "Original" analysis, using the outbreak definition of 1 or more cholera cases being reported in a specific week.
